# Nature’s Secret Neuro-Regeneration Pathway in Axolotls, Polychaetes and Planarians for Human Therapeutic Target Pathways

**DOI:** 10.3390/ijms252211904

**Published:** 2024-11-06

**Authors:** Nur Izzati Mansor, Tengku Nabilatul Balqis, Mohd Nizam Lani, Kwan Liang Lye, Nor Azlan Nor Muhammad, Wan Iryani Wan Ismail, Shahidee Zainal Abidin

**Affiliations:** 1 Department of Nursing, Faculty of Medicine, Universiti Kebangsaan Malaysia, Cheras 56000, Kuala Lumpur, Malaysia; nurizzatimansor@ukm.edu.my; 2 Faculty of Science and Marine Environment, Universiti Malaysia Terengganu, Kuala Nerus 21030, Terengganu, Malaysia; tengku.nabilatul.balqis@gmail.com (T.N.B.); waniryani@umt.edu.my (W.I.W.I.); 3 Faculty of Fisheries and Food Science, Universiti Malaysia Terengganu, Kuala Nerus 21030, Terengganu, Malaysia; nizamlani@umt.edu.my; 4 ME Scientifique Sdn Bhd, Taman Universiti Indah, Seri Kembangan 43300, Selangor, Malaysia; elson@mescientifique.com; 5 Institute of Systems Biology (INBIOSIS), Universiti Kebangsaan Malaysia, Bangi 43600, Selangor, Malaysia; norazlannm@ukm.edu.my; 6 Research Interest Group Biological Security and Sustainability (BIOSES), Faculty of Science and Marine Environment, Universiti Malaysia Terengganu, Kuala Nerus 21030, Terengganu, Malaysia

**Keywords:** neuro-regeneration, axolotl, polychaete, planarian, mammalian

## Abstract

Despite significant improvements in the comprehension of neuro-regeneration, restoring nerve injury in humans continues to pose a substantial therapeutic difficulty. In the peripheral nervous system (PNS), the nerve regeneration process after injury relies on Schwann cells. These cells play a crucial role in regulating and releasing different extracellular matrix proteins, including laminin and fibronectin, which are essential for facilitating nerve regeneration. However, during regeneration, the nerve is required to regenerate for a long distance and, subsequently, loses its capacity to facilitate regeneration during this progression. Meanwhile, it has been noted that nerve regeneration has limited capabilities in the central nervous system (CNS) compared to in the PNS. The CNS contains factors that impede the regeneration of axons following injury to the axons. The presence of glial scar formation results from this unfavourable condition, where glial cells accumulate at the injury site, generating a physical and chemical barrier that hinders the regeneration of neurons. In contrast to humans, several species, such as axolotls, polychaetes, and planarians, possess the ability to regenerate their neural systems following amputation. This ability is based on the vast amount of pluripotent stem cells that have the remarkable capacity to differentiate and develop into any cell within their body. Although humans also possess these cells, their numbers are extremely limited. Examining the molecular pathways exhibited by these organisms has the potential to offer a foundational understanding of the human regeneration process. This review provides a concise overview of the molecular pathways involved in axolotl, polychaete, and planarian neuro-regeneration. It has the potential to offer a new perspective on therapeutic approaches for neuro-regeneration in humans.

## 1. Introduction

Neuro-regeneration is a fascinating and complex field of study that revolves around the ability of the nervous system to repair and regenerate itself. This impressive capacity for neuro-regeneration is shown in several organisms, such as axolotls, polychaetes, and planarians, and it could offer valuable insights for researchers striving to understand and harness these potential therapeutic applications in humans.

Unlike these organisms, the human nervous system faces significant challenges regarding self-repair, especially in the central nervous system (CNS). The CNS environment becomes inhibitory to axonal regrowth after injury due to the formation of inhibitory glial scars and the lack of supportive growth factors [1]. The nervous system is an intricate and essential component of organisms, serving as a central hub for controlling and coordinating a wide range of functions and activities. The activities include transmitting signals between various body parts and processing sensory information. The nervous system comprises the CNS and the peripheral nervous system (PNS). The CNS encompasses both the brain and the spinal cord. The brain serves as the central hub of the human body, overseeing the intricate processes of information processing, decision-making, and regulating diverse physiological activities. The spinal cord is an intermediary connecting the CNS, specifically the brain, with the PNS, which encompasses the body’s various organs and tissues.

While humans struggle with limited neuro-repair, some organisms have remarkable neuro-regenerative abilities. One of these organisms is known as the axolotl, a type of aquatic salamander native to Mexico which is famous for its impressive regenerative capabilities [2,3,4]. Axolotls can regenerate entire limbs, including the nerves, spinal cord, and other tissues [5]. One of the most striking features of axolotls is their capacity to regenerate portions of the spinal cord. Following injury, axolotls can restore structure and function to the spinal cord, allowing them to regain motor control and sensory functions [6]. Their ability to regrow complex structures like limbs and spinal cords makes them valuable for understanding the mechanisms involved in neural repair.

Moreover, polychaetes are marine worms known for their segmented bodies and diverse habitats. Polychaetes have a more straightforward nervous system than vertebrates, making them more accessible for studying the fundamental processes of neuro-regeneration [7]. Interestingly, their capacity to regenerate both the central and peripheral nervous systems can serve as a model for comprehending neuro-regeneration mechanisms [8]. Another worm, the planarian, also has a remarkable regenerative nervous system capacity [9]. These flatworms can be found in various aquatic environments and are characterised by their ability to regenerate their bodies [10]. The presence of this organism’s pluripotent stem cells, known as neoblasts, can give rise to various cell types, including neurons [11]. As a result, it can restore complex structures, including the cephalic ganglia, and re-establish functional connections of neural circuits.

Collectively, these model organisms provide distinct advantages, enabling researchers to delve into the complexities of nerve cell regeneration, neural circuit reconstruction, and the involvement of stem cells. Therefore, in this review, we highlight the molecular mechanisms behind the process of neuro-regeneration, focusing on four different organisms: axolotl, polychaetes, planarians, and mammalians. In addition, we also discuss the current research regarding human neuro-regeneration and its limitations. The extrapolation of the neuro-regeneration process in these organisms (axolotls, polychaetes, and planarians) has the potential to yield significant insights for developing therapeutic approaches aimed at augmenting the regenerative capabilities of the human nervous system.

## 2. Regenerative Capabilities of Axolotls, Polychaetes, and Planarians

Axolotls, polychaetes, and planarians demonstrate exceptional neuro-regenerative abilities that exceed those observed in humans, which is attributed to their unlimited stem cell capacity. The axolotl demonstrates the ability to regenerate complete limbs and portions of its CNS via a mechanism that entails the dedifferentiation of stem cells, succeeded by proliferation and redifferentiation to create new tissues [12]. Interestingly, axolotls possess the ability to regenerate both the structure and function of injured tissues without the formation of scar tissue, a major impediment to regeneration in humans [13]. The activation of neural progenitor cells (NPC) is central to this process, as these cells proliferate and differentiate into diverse neural cell types, encompassing neurons and glial cells [12]. Axolotls exhibit the ability to regenerate neurons and direct axons to establish functional synapses following spinal cord and brain injuries, facilitating complete restoration of motor and sensory functions [14]. The regeneration of limbs in axolotls is intricately linked to nerve regeneration, as the regrowth of nerves delivers critical growth signals necessary for the reformation of limb tissues [15].

In polychaetes, neuro-regeneration depends on two primary processes: stem cell activation and transdifferentiation [16]. Following injury, neural stem cells in the CNS, especially near the ventral nerve cord (VNC), are activated. The cells undergo proliferation and differentiation into neurons, glial cells, and other supportive tissues to restore the damaged neural structures. Transdifferentiation is essential for neuro-regeneration alongside stem cell activation [17]. This process entails the direct transformation of non-neural cells, such as epithelial or mesodermal cells, into neurons or glial cells, facilitating a swift response to injury. Growth factors, such as fibroblast growth factor (FGF), facilitate this reprogramming, enhancing regeneration by converting adjacent cells into functional neural components [18]. The interaction between stem cell activation and transdifferentiation establishes an efficient mechanism for neural repair in polychaetes. Stem cells produce new neural cells, whereas transdifferentiated cells aid in regeneration, facilitating the re-establishment of intricate neural networks without the development of scar tissue, which generally hinders regeneration in mammals.

Planarians exhibit a significant abundance of pluripotent stem cells, known as neoblasts, capable of differentiating into diverse cell types, such as neurons [19]. Upon injury, a planarian initiates a localised signalling cascade characterised by the involvement of growth factors and alterations in specific gene expression, which subsequently activate neoblasts at the site of injury. Neoblasts migrate to the damaged region, proliferate, and differentiate into the requisite cell types, thereby restoring lost structures. This process involves the regeneration of the CNS, wherein planarians are capable of reconstituting complex neural networks, including brain-like structures referred to as cerebral ganglia [20]. Planarians demonstrate the capacity to regenerate particular types of neurons and synaptic connections, which is essential for the restoration of function. Studies indicate that specific molecular pathways, including Wnt, bone morphogenetic protein (BMP), and Hedgehog signalling, are essential in the regulation of regenerative processes.

In comparison, humans exhibit a restricted ability for regeneration within the nervous system. Humans have neural stem cells located in certain brain regions, such as the hippocampus. These cells are multipotent, exhibiting a limited capacity to produce new neurons or glial cells. Additionally, glial scar formation presents a substantial obstacle to axon regrowth following neural injury in humans. Investigating the significant cellular plasticity in axolotls, polychaetes, and planarians, along with their capacity to prevent inhibitory scar formation, provides critical insights that may enhance therapeutic approaches to address the challenges of neuro-regeneration in humans.

## 3. Limitation of Nervous System Regeneration in Humans

**Figure 1:**
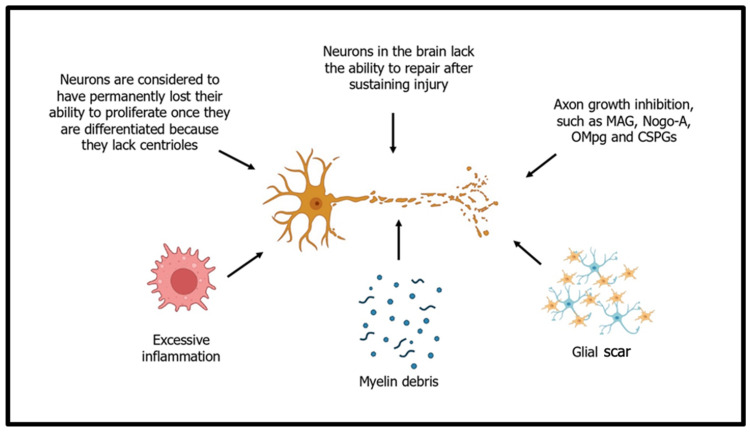
The factors limiting human nerve regeneration.

The human nervous system faces formidable challenges when it comes to self-repair. Both the CNS and PNS exhibit limited regenerative capacities due to various complex factors (Figure 1). Neurons, the basic building blocks of the nervous system, have limited capacity for cell division and mitotic abilities, limiting their capacity for regeneration due to a lack of cell proliferation in human neurons [21]. This cell typically does not undergo mitosis, in contrast to other cell types, to replace damaged or dead cells [22]. Neurons generally cease cell division during development and remain non-dividing throughout an individual’s life [23]. Moreover, the damaged neurons cannot be restored and regenerated; however, part of the damaged nervous system can be reorganised in response to any injury.

One reason why injured neurons are unable to repair is due to repair infidelity. Instead of the neuron being repaired, it undergoes cellular senescence. This is based on a previous study that showed that defects in DNA repair lead to cell cycle-induced senescence in neurons after prolonged alcohol consumption [24]. The alterations are due to an accelerated process of ethyl alcohol oxidation in these cells through the NADPH-dependent cytochrome pathway. This interferes with the folate metabolism of the 1-carbon network, which aids in selecting the DNA repair pathway through non-cell cycle-dependent mismatch repair networks. The malfunction leads to the reactivation of the less favoured cell cycle-dependent homologous recombination (HR) repair in post-mitotic cells, causing them to undergo a process similar to the cell cycle. Mature neurons are no longer capable of cell division. Thus, these cells are halted at checkpoints rather than completing a full round of the cell cycle required for HR-mediated repair. The persistence of repair intermediates leads to the nuclear build-up of p21 and cyclin B, which triggers persistent cell cycle exits and irreversible senescence response [24].

Repair infidelity in neurons due to DNA damage can result from a variety of causes beyond alcohol consumption, including age-related degeneration, traumatic injuries, and neurodegenerative diseases. A previous study showed that defective transcription-coupled repair (TCR) expedites neurodegeneration in a *C. elegans* model for Cockayne syndrome (CS), therefore emphasising the primordial significance of DNA damage in promoting age-related neuronal disease [25]. Furthermore, age-related degeneration results in decreased activity of DNA repair pathways like base excision repair (BER) and non-homologous end joining (NHEJ), leading to persistent DNA lesions that impair neuron function and contribute to cognitive decline. This condition has been observed in Alzheimer’s disease (AD) and Parkinson’s disease (PD), both of which are linked to DNA damage and altered expression and activity of DNA repair genes, including reduced effectiveness of nucleotide excision repair [26,27]. Various DNA repair mechanisms, including mismatch repair, play a role in the repeat expansion linked to Huntington’s disease (HD), whereas mutant huntingtin has been associated with impairments in repairing transcription-induced DNA strand breaks [28].

The CNS contains inhibitory factors that hinder the regeneration of axons. These factors include myelin-associated inhibitors such as Nogo, MAG, OMgp and chondroitin sulphate proteoglycans [29]. In addition, glial scar formation is a phenomenon that occurs in response to CNS injuries and is characterised by the accumulation of glial cells at the site of injury [30]. Glial cells are highly flexible and responsive to environmental changes, displaying varying functions and roles. Completely removing the glial scar may not promote spinal cord regeneration because the glial scar serves a dual function. The glial scar is crucial in restricting the propagation of inflammation and the harmful impact of fibrotic tissue and macrophages [31]. Nevertheless, it also acts as a physical barrier that impedes axon regeneration [32]. Another factor that hinders the regrowth of neurons is inflammatory cells. During spinal injury, the inflammatory cells release cytokines and growth factors such as NGF, VEGF, TNF-α, and IL-1, contributing to the inhibitory environment and subsequently impeding neuronal growth [33].

## 4. The Mechanisms of Tissue Regeneration

Organisms such as axolotls, polychaetes, and planarians are known for their remarkable ability to regenerate cells, tissues, and entire body parts. Studies have used advanced technology, including sequencing and various molecular tools, to explore the mechanisms behind this extraordinary capability. The findings suggest that there are conserved mechanisms and processes involved in tissue remodelling and regeneration between these organisms. Here are some pathways identified in these organisms that have similarities with the mechanisms found in humans.

### 4.1. Axolotls

Axolotls are salamanders that play a crucial role as a model organism for understanding the development, regeneration, and evolution of tetrapods. Most studies have used Mexican axolotls (*Ambystoma mexicanum*) as a research model due to their extensive molecular toolkits. These techniques have made it easier to identify the specific cells responsible for regeneration and the molecular pathways that control the process. For instance, a previous study showed NPC distribution in response to injury in an axolotl’s brain. In this study, removing a large telencephalic tissue activated endogenous NPCs in the ventricular zone. These cells spontaneously proliferate, producing progeny that migrate throughout the telencephalon region before differentiating into neurons. These progenitor cells are crucial in telencephalon regeneration after tissue removal [34].

**Figure 2:**
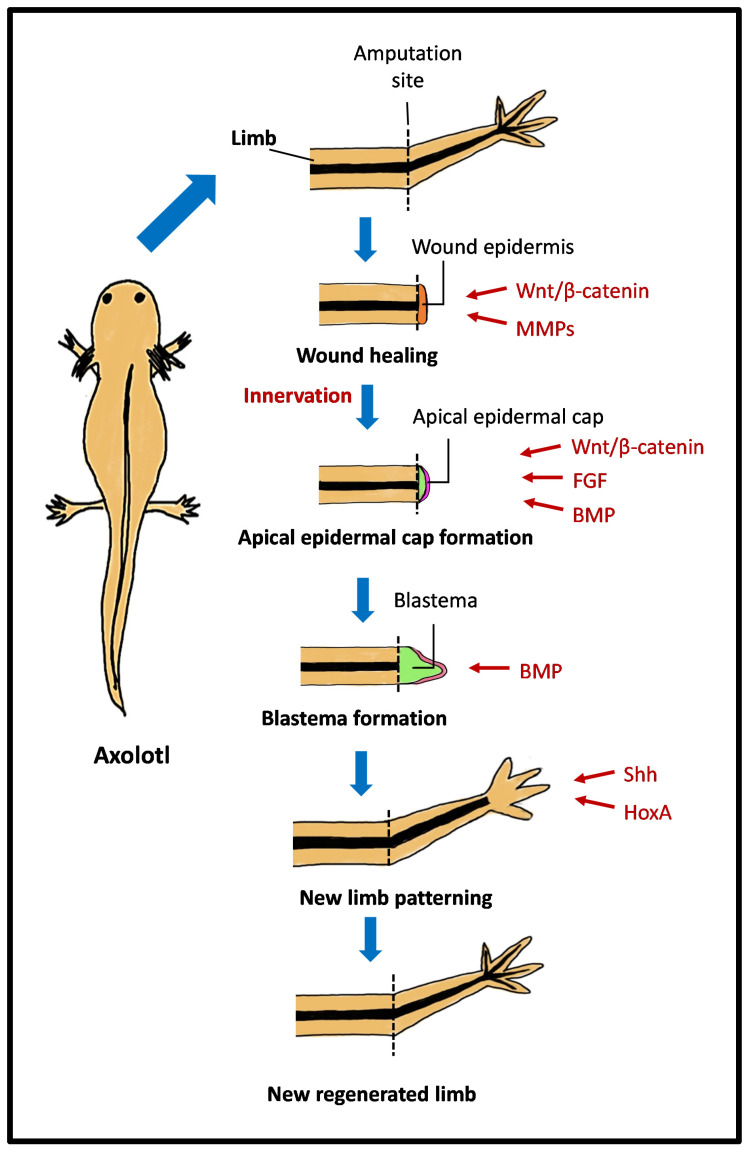
Limb regeneration in the axolotl. A step-by-step process involving wound healing, apical epidermal cap formation, blastema development, and limb patterning. Key molecular signals, including Wnt/β-catenin, MMPs, FGF, BMP, Shh, and HoxA, guide each stage, culminating in the formation of a fully regenerated limb.

Another study found that upon a large mechanical injury to the pallium, the adult axolotl can regenerate the original neuronal diversity [35]. Newborn neurons acquire intrinsic electrophysiological properties and process afferent input in a manner indistinguishable from endogenous neurons in uninjured brains. However, axolotls possess limited capacity to rebuild original tissue architecture after large deletions of the pallium. The processes and mechanisms that initially trigger wound closure and sustain brain repair in regenerative vertebrate species are unknown. The study found that early steps of wound closure involve generating thinner processes from a stump that directionally grow towards each other before fusing. This strategy resembles that observed in the limb, where closure of the wound by the wound epithelium after amputation is necessary for subsequent regenerative steps (Figure 2).

During the limb regeneration process, an axolotl regenerates by generating a specialised structure known as a blastema, followed by cellular rearrangement to develop the new functional tissues [15,36,37]. A nerve supply, the epithelium, and connective tissue cells are required for blastema formation and growth [12]. Within hours following limb injury, a wound epithelium migrates to the injury sites and covers the wound area. The wound epithelium becomes innervated within days and transforms into an apical epithelial cap (AEC). During this transition, the basal keratinocytes shift to a non-proliferative state, signalling and recruiting connective tissue fibroblasts to generate the early blastema. At later stages of development, the cells in the blastema’s basal region begin to differentiate, while the cells at the blastema’s apical tip remain proliferative and undifferentiated. The blastema cells continuously and progressively differentiate into limb tissues from the blastema’s basal to apical regions [12,15,38,39].

Several signalling pathways and transcription factors have been associated with limb regeneration and patterning. Modulation of the Wnt/β-catenin signalling pathway also plays a vital role in the process of limb regeneration in axolotls, including the regulation of nerve innervation and skeletal morphogenesis [40,41,42]. A previous study documented that a reduction in Wnt and BMP signalling during limb regeneration in axolotls leads to changes in the formation of the apical ectodermal cap. These changes impede normal limb regeneration in axolotls. However, injecting a virus (Ad-Axin1) that inhibits Wnt signalling after amputation resulted in spike-like limbs without digits. This suggests that the inhibition of Wnt signalling impairs the axolotl’s ability to regenerate normal limbs [42]. Another study examined Wnt signalling genes’ expression patterns in developing and regenerating axolotl limbs [40]. They observed that Wnt3a was highly expressed in the epithelium in both the dorsoventral and anteroposterior axes, Wnt5a showed high expression in the distal basal epithelium and mesenchyme, and Wnt5b was found mainly in the distal mesenchyme.

In addition to Wnt/β-catenin signalling, FGF and BMP signalling pathways are essential in the regeneration process, particularly in blastema formation. FGF signalling and sonic hedgehog (Shh) signalling govern the anterior–posterior patterning of regenerating axolotl limbs [43]. During axolotl limb regeneration, the wound epithelium of the amputated limb secretes FGF-8, which is utilised by the underlying blastemal cells and triggers mitotic activity. These blastemal cells subsequently secrete FGF-10, which initiates a positive feedback loop that stimulates the regeneration of the surrounding epithelial cells [44,45,46]. It is assumed that FGF2 and FGF8 influence the wound epithelium and lead to the formation of the AEC. Together with the nerves, the AEC creates a regeneration environment that inhibits normal wound healing while promoting limb regeneration through blastema formation [47].

Similarly, BMPs are key nerve factors that regulate anterior–posterior and proximo-distal skeletal patterning and blastema development [48]. BMP signalling is involved in cell condensation and apoptosis in regenerating axolotl limbs [49]. Meanwhile, Shh signalling is required for the proliferation, survival, and extension of forelimb field progenitor cells to develop the axolotl forelimb bud. As the limb bud emerges from the flank, Shh signalling activates FGF8 and downstream FGF signalling targets in the limb mesoderm, supporting cell proliferation at the distal tip of the limb bud [43,50,51] and suppressing Shh signalling using cyclopamine during axolotl limb regeneration, resulting in digit loss. Loss in the most posterior digits is caused by late inhibition, whereas loss in the more anterior digits is caused by early inhibition. This highlights the crucial role of Shh signalling in the anterior–posterior patterning of axolotl limb regeneration [52,53].

Additionally, Hox genes also play critical roles in the development and regeneration of axolotls [54,55]. Several studies have shown that Hox genes regulate expression patterns during axolotl brain and spinal cord development and regeneration [54,55,56,57]. For example, the HoxA9 and HoxA13 genes are expressed in developing limb buds and regenerating blastema cells. Hox A9 is expressed early in the entire limb bud, while Hox A13 is expressed later in the distal-most part of the limb bud, as seen in other vertebrates, such as mice and chicks [57]. Both genes start their expression in the same population of stump cells, and these genes reactivate within 24 h of amputation, suggesting that re-expression may occur concurrently rather than sequentially over time.

In the early stages of blastema formation, HoxA9 and HoxA13 are colocalised in both the proximal and distal regions of the limb. As regeneration progresses, the expression patterns of HoxA9 and HoxA13 differ along the proximal–distal axis. HoxA9 is expressed in the proximal region during blastema growth, whereas HoxA13 is only expressed in the distal region. In the later stages of regeneration, the relationship between HoxA expression patterns is the same as in the developing limb, with HoxA13 expression associated with regeneration of the hand and HoxA9 expression associated with regeneration of the hand, forearm, and distal humerus [57]. In addition to that, Hoxb13 and Hoxc10 are highly expressed in the spinal cord during tail regeneration. These high expression levels are also evident in the regenerating hind and forelimbs [58].

The cellular and molecular mechanisms in axolotl regeneration provide valuable insights into the mechanisms of human tissue regeneration. For example, a mixture of human growth factors found in axolotls such as Bmp2, Fgf2, and Fgf8 can functionally replace nerve signalling to promote the development of an ectopic blastema and support later stages of limb regeneration [59]. Moreover, the key pathways in axolotls, like Wnt, BMP, and FGF, allow mature cells to revert to a stem cell-like state, promoting cellular plasticity and allowing us to extrapolate these pathways as potential targets for enhancing human tissue repair. Furthermore, another study demonstrated limited neutrophil infiltration and a delay in extracellular matrix (ECM) production, all of which could contribute to scar-free healing in axolotls and minimise fibrosis [13]. These findings may provide useful information on how to prevent scar tissue formation during wound healing processes in humans. This study also further suggests that matrix metalloproteinases (MMPs) play an important role in the migration and regeneration of keratinocytes, with prolonged activity potentially regulating the rate of ECM deposition [13]. In addition, the cell that surrounds the blastema consists of several multipotent progenitor cells from different tissues, each of which plays a lineage-specific role in limb tissue regeneration [60]. Thus, by investigating the role of stem cells within the blastema, the researchers hope to better understand how the human response to limb amputation can be modulated. This could lead to the regeneration of functional limbs.

### 4.2. Polychaetes

Bristle-worms, also known as polychaetes, are annelids that live in marine environments. They can be found in various habitats, ranging from the shallow coastal zone to the deepest depths. They occupy both soft and stony bottoms, as well as lead an aquatic lifestyle. These animals serve as a valuable model for regeneration studies due to their relatively simple anatomy, characterised by a series of comparable segments extending from the posterior pygidium to the anterior prostomium. Furthermore, in many species, the process of regenerating identical body parts is regulated in distinct ways based on numerous characteristics, such as body polarity.

**Figure 3:**
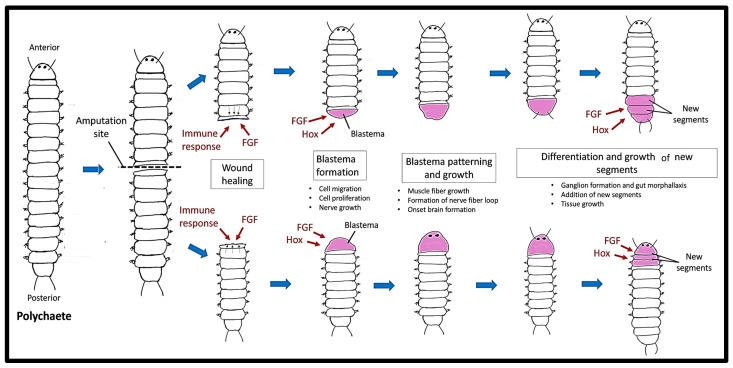
Regeneration process in polychaetes. Following amputation, an immune response is initiated, and fibroblast growth factor (FGF) stimulates wound healing. This leads to the formation of a blastema, where cell migration, cell proliferation, and nerve growth occur. The blastema undergoes patterning and growth, involving muscle fibre development, nerve fibre loop formation, and early brain development. Finally, differentiation and segmental growth lead to the addition of new body segments, with ganglion formation, gut morphallaxis, and tissue expansion, orchestrated by FGF and Hox gene expression.

Most polychaetes regenerate through epimorphosis, which involves cell proliferation and the development of a blastema [16,61,62]. Generally, polychaete regeneration is divided into five stages following amputation (Figure 3): (1) wound healing, (2) blastema development, (3) blastema patterning and growth, (4) differentiation of the first regenerated segment, and (5) development and differentiation of the subsequent 5–6 segments. On average, 10 days after amputation (dpa) is required to restore the normal anatomy of the posterior body end [62]. During wound healing, substantial cell migration to the wound site occurs immediately after amputation, followed by tissue re-epithelialization. The migration of cells to the wound site is facilitated by rapid muscle contraction. Migrating cells generate the wound plug by clotting and activating an innate immune response. The re-epithelialization process occurs through cell rearrangement without the involvement of mitosis. The epidermis of the regenerated body wall initiates dedifferentiation and cell division, resulting in blastema development. Similar to the axolotl, the polychaete blastema undergoes patterning and growth processes to restore new tissues or segments [16,63,64].

The regeneration of the nervous system following amputation involves the activation of adjacent tissues and the proliferation of newly generated cells in the regenerative bud. Neurites originating from the VNC and the PNS extend into the wound site and infiltrate the wound epithelium, creating a network of nerves around the intestine [62,65]. Once the regeneration blastema emerges, the neurites may have a varying number of nerves that are either thicker or thinner, ultimately ending at the location where pygidial or prostomial appendages are formed [66]. The lateral nerves of the VNC frequently form a loop, which is characteristic of both anterior and posterior regeneration [67]. The neural precursor cells of the regenerative bud concentrate around the initially developed neuropil, potentially entering from the outermost layer of the bud.

Similar to humans, polychaetes have an innate immune system; however, this invertebrate lacks adaptive immunity. To date, the regulation of immune response-related pathways during polychaete regeneration remains poorly understood. However, previous studies have described how coelomocytes, a specialised immune cell, migrate to the wound site in the early stages of polychaete regeneration and phagocytize cellular debris, such as damaged epithelia and muscle cells [16,64]. These coelomocytes form a “wound plug” that acts as a mechanical barrier against further fluid loss [68]. During the study of another class under phylum Annelida, *Eisenia andrei* earthworms, it was experimentally found that the reduction of coelomocytes reduces cell proliferation in both the anterior and posterior blastema. This suggests that coelomocytes may be essential for tissue regeneration [69].

The entire pre-existing nervous system undergoes significant rearrangements, with the process of neural morphallaxis being extensively explained using evidence from the regeneration of Lumbriculus variegatus [70]. The neuronal networks undergo rapid alterations in the initial days following an injury, resulting in the reorganisation of sensory fields to align with their new location in the body [16]. The investigation of the molecular mechanisms underlying brain regeneration in polychaetes has been limited. The most pronounced differential expression is observed in neurodifferentiation markers, including glutamine synthetase, slit, elav, neurofilament NF70, and nerve growth factor.

An early activation of FGF expression was detected after amputation and before cell proliferation started. This suggests that FGF plays a role in blastema initiation during regeneration. The epidermis and inner cells were found to produce FGFs during the early transcriptional response and recruit FGFR-positive mesodermal and ectodermal cells to trigger blastema proliferation and development [18]. This early FGF activity drives cell proliferation, migration, and patterning at the wound site, facilitating the growth of a regeneration bud that will ultimately restore the lost structure. This study also found that the inhibition of FGF signalling suppresses cell proliferation and prevents the development of a blastema and regeneration bud. Furthermore, the FGF was expressed in the late stages of regeneration in the ectoderm and nervous system of newly formed segments, suggesting that FGF plays a role in axis elongation and patterning.

Hox genes have also been reported to be involved in restoring the anterior–posterior axis after amputation in polychaete. During post-larval development and regeneration, the Hox genes provide positional information but do not influence segmental diversity. In the initial regeneration phase (before 48 h), Hox expression patterns are reorganised and restored within the new body boundaries. The boundaries of Hox gene expression shift in the aged tissues within 18 h before blastema development. From 24 h post amputation (hpa) onward, Hox genes are highly expressed during blastema development and remain active in the rudimentary terminal structures for up to 3 dpa when they become morphologically distinct. Interestingly, Hox1-5, Hox7, Lox4, Lox2, Post2, and Cad are expressed on day 7, when new segments are formed [71].

### 4.3. Planarians

**Figure 4:**
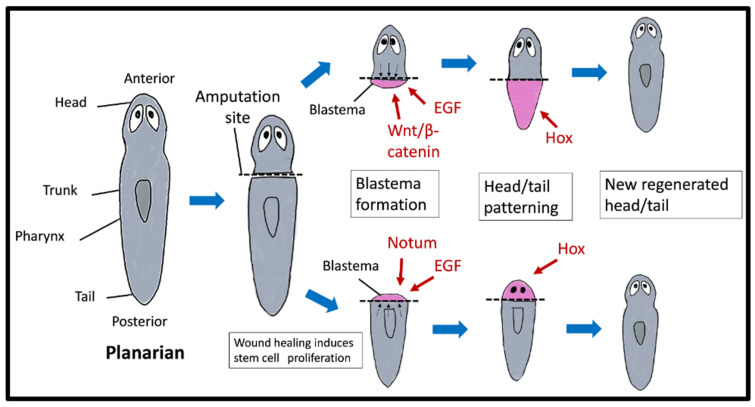
Regeneration process in planarians. Following amputation, wound healing triggers stem cell proliferation, leading to blastema formation. Key signalling molecules, including Wnt/β-catenin, EGF, Notum, and Hox genes, direct the patterning of the new head or tail, resulting in a fully regenerated structure that restores anterior–posterior polarity.

Planarians belong to the phylum Platyhelminthes and are often found in freshwater streams and ponds. Planarians have the ability to regenerate and repair substantial portions of their body structures that have been lost or damaged. Regeneration occurs in three phases (Figure 4): (1) the closure of the wound, which takes place within the first 30–45 min, (2) the development of a cell mass called a blastema at the site of injury within 48–72 h, and (3) the repositioning of old and new tissue over the next 1–2 weeks, leading to the restoration of the worm’s normal morphology. The extraordinary regenerative capacity of planarians is attributed to their adult stem cell population, the neoblasts, which are found throughout the body. In response to injury, the neoblasts migrate to the wound site, differentiate into tissue-specific progenitor cells, and form a blastema [72,73,74]. The neoblasts of planarians show two distinct peaks of mitosis after amputation. The first peak involves migrating neoblasts that respond to wound healing within 6 h, and the second peak targets wound sites after significant injury within 48–72 h to promote blastema formation [73]. The increase in proliferation rate is influenced by an initial local increase in apoptosis at the wound site [75]. The blastema is remodelled to restore symmetry and proportions (morphallaxis) [74].

Several signalling pathways play a role in regulating neoblast proliferation and the anterior–posterior polarity of the planarian blastema [76]. Following amputation, neoblasts begin self-renewal and differentiation. These processes are reported to be regulated by the EGF signalling pathway [77,78,79]. Fraguas et al. (2011) discovered three EGF receptors in planarians named Smed-egfr-1, Smed-egfr-2, and Smed-egfr-3. Smed-egfr-1 is primarily involved in stem cell proliferation and differentiation, making it critical for generating the cells needed to replace lost tissues [77]. Smed-egfr-2 has a role in the differentiation and organisation of these cells, ensuring that they reach the correct locations and form coherent structures. Smed-egfr-3, meanwhile, is thought to influence neoblast repopulation, cell proliferation, and asymmetric cell division [79]. These receptors likely interact through sequential and overlapping signalling pathways, creating a coordinated cascade that ensures efficient and accurate tissue regeneration. Targeting specific EGFRs could theoretically improve regeneration for particular tissues or injuries in planarians. For instance, enhancing Smed-egfr-1 activity might increase stem cell availability, potentially speeding up the regeneration process, whereas inhibiting Smed-egfr-2 could modulate cell migration to prevent overgrowth or disorganised tissue formation [77]. Thus, selective modulation of these EGFRs could offer strategies to enhance or refine regenerative processes in planarians and may eventually inspire approaches to control tissue repair in other organisms, including humans.

Tissue regeneration in planarians also depends on the Wnt and BMP signalling pathways. The Wnt signalling pathway is crucial in regulating the polarity of planarians’ primary body axes (anterior–posterior and dorsal–ventral). Several studies have shown that silencing β-catenin leads to the regeneration of heads (anterior region) instead of tails (posterior region) [80,81]. In addition, the distribution of Wnt-related genes in planarians is closely linked to the anterior–posterior axis, with different expression patterns between the anterior and posterior sites. For example, Smed-wntP-1, Smed-wntP-2, Smed-wnt11-1, and SmedFz-d are expressed in the posterior region, Smed-wnt2-1 is expressed in the pre-pharyngeal region, and Smed-sFRP-1 is expressed in the anterior region [80,81]. The introduction of RNAi targeting a single β-catenin (Smedβ-catenin-a), both Dishevelled homologs (SmedDvl-a/b), or APC (SmedAPC) resulted in significant changes in the anterior and posterior sites of regenerating tissues [81]. The experiment was carried out by dividing the planarian in two and subjecting it to RNAi. As a result, the anterior marker (Smed-sFRP-1) was expressed in the posterior head of Smedβ-catenin-a and SmedDvl-a/b RNAi worms. However, the posterior marker (Smed-wntP-1, Smed-wntP-2, Smed-wnt11-1, and SmedFz-d) was significantly decreased or completely absent. In contrast, the posterior marker was expressed in the anterior tail of the SmedAPC RNAi worm, while the anterior marker exhibited significant reduction or absence. The RNAi-treated animals exhibited incomplete regeneration of heads and tails, indicating that β-catenin regulators play a crucial role in the early stages of regeneration [81].

In addition, the BMP signalling pathway has been identified as a regulator of the planarian dorsal–ventral axis of planarians. BMP signalling was involved in developing new tissues at the regeneration midline, in the dorsal–ventral patterns in new tissues, and in maintaining the dorsal–ventral pattern in adult tissue during homeostasis. The gene smedbmp4-1 is frequently expressed along the dorsal midline before forming a blastema [82]. The absence of components of the BMP signalling pathway, such as bmp4, smad1, smad4, or tolloid, can lead to an aberrant dorsal–ventral polarity [83,84]. A recent study revealed that the dorsoventral regulator BMP4 in planarians plays a crucial role in controlling the anteroposterior axis by repressing the posterior wnt1 gene. Inhibition of BMP4 and smad1 increased anterior expression of wnt1 while suppressing nog1 and nog2 using RNAi-increased BMP signalling and decreased wnt1 expression. The changes in BMP signalling significantly affected anteroposterior (head-to-tail) body axis patterning, as evidenced by the changes in Wnt gene expression. However, these changes did not affect head regionalisation. The suppression of bmp4 expression also resulted in the medial expansion of the lateral determinant wnt5 and decreased expression of the medial regulator slit. Simultaneous silencing of BMP4 and wnt5 resulted in lateral ectopic eye phenotypes, suggesting that BMP4 regulates wnt5 activity in mediolateral axis patterning [85]. Collectively, the Wnt signalling pathway regulates the anteroposterior axis of the planarian, while the Bmp signalling pathway regulates the dorsoventral axis.

Several Hox genes also play critical roles in the asexual reproduction and regeneration process in planarians [36,86]. Currie et al. discovered that the 13 HOX genes (HOX 1-13) of Schmidtea mediterranea are distributed throughout the genome and not clustered together [36]. Five genes are expressed axially, two are expressed radially across the body margin, and the remaining are tissue-specific. They also found that planarians exhibit the most anteriorly restricted expression of the putative HOX3 homolog (Smed-Hox3b), which is significantly enriched in the neck region [36]. Hox genes and genes encoding the transcription factor SP5 were expressed in many tissue types. They showed the earliest detectable changes during regeneration following the early stages of wound-induced gene expression. It has been demonstrated that post-2d, a posterior Hox gene of planarians, is essential for normal tail regeneration. In contrast, SP5 suppresses the expression of genes associated with the trunk region in the tails of planarians and promotes distinct tail–trunk–body domains [87]. Another study has reported that inhibition of β-catenin-1 resulted in the downregulation of Hox genes (Post-2c, lox5a, Post-2d, and hox4b) in the posterior region. Inhibition of Post-2d and Hox genes led to impaired tail regeneration, while inhibition of SP5 upregulated the expression of trunk genes in the tail of planarians [87]. This shows that Wnt signalling and HOX genes play a role in the patterning and growth of the posterior of planarians.

## 5. Molecular Mechanisms in Mammalian Nerve Regeneration

**Figure 5:**
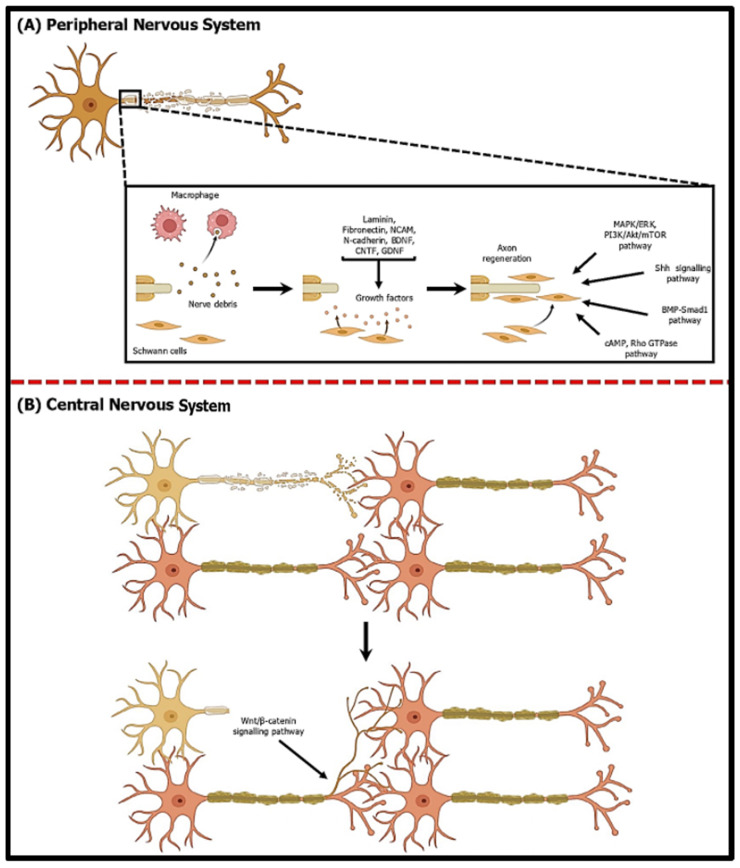
Comparison of neuro-regeneration mechanisms in the peripheral and central nervous systems. (**A**) In the peripheral nervous system, when a nerve is damaged, macrophages clear nerve debris and Schwann cells produce various growth factors like laminin, fibronectin, NCAM, N-cadherin, BDNF, CNTF, and GDNF. These factors facilitate axon regeneration through several signalling pathways, including the MAPK/ERK, PI3K/Akt/mTOR, Shh, BMP-Smad1, and cAMP/Rho GTPase pathways. This results in successful axon regrowth and functional recovery. (**B**) In central nervous system, the Wnt/β-catenin signalling pathway promotes the branching of axons and dendrite extensions, thus allowing existing neurons to form new connections.

Mammalians, particularly humans, have a limited ability to regenerate their neurological system. While repairing the nervous system is generally successful in the PNS, it is less effective in the CNS. The source of this difference does not seem to originate from the neurons themselves but, rather, from the glial cells present in the PNS compared to those present in the CNS [88]. The PNS and CNS have distinct environments primarily because Schwann cells in the PNS provide myelin protection to neuronal axons, whereas oligodendrocytes in the CNS ensheathe the neurons. Therefore, there are differences between nerve regeneration in the PNS and CNS (Figure 5).

### 5.1. Peripheral Nerve Regeneration

Schwann cells play a significant role in PNS regeneration by providing favourable conditions. This also includes CNS neurons that have axons extending into the PNS. The Schwann cells regulate and release various extracellular matrix proteins, such as laminin and fibronectin [89]. These proteins are crucial in creating a pathway to regress injured axons. Although these extracellular matrix proteins indicate the route for axonal regeneration, these axons need supplementary components to assist in pathfinding to successfully regenerate across an injured area. To address this issue, Schwann cells increase the production of several cell adhesion proteins, such as neural cell adhesion molecules (NCAMs) and N-cadherin from the cadherin family [90]. The Schwann cells express cell adhesion proteins that bind to the adhesion proteins expressed by the axonal growth cone. This allows the growth cones to travel through the injury site and establish correct synaptic connections with target cells.

Schwann cells can also generate and release different neurotrophic factors, such as brain-derived neurotrophic factor (BDNF), ciliary neurotrophic factor (CNTF), and glial-derived neurotrophic factor (GDNF), in response to injury [91]. These neurotrophic substances are likely present to start a regeneration process, resulting in the development of a growth cone and the initiation of pathfinding inside this growth cone. BDNF promotes neuron survival, axonal outgrowth, and synaptic plasticity, creating an environment that fosters neuronal health and structural repair [92]. CNTF supports axonal elongation and differentiation, and GDNF enhances Schwann cell myelination and regenerating axons [93,94]. These factors often work synergistically, with each one activating distinct but complementary signalling pathways to amplify regenerative signals.

In addition, the profiles of neurotrophic factors in denervated Schwann cells vary in magnitude and pattern depending on the central–peripheral location and modality of the associated axons. For example, GDNF is mainly upregulated in the dorsal and ventral roots, whereas BDNF is the most notable of the factors upregulated by denervated sensory Schwann cells in the peripheral nerve. The expression of CNTF was not modality- or site-specific. The expression patterns of selected neurotrophic factor genes in the motor and sensory phenotypes for 5, 1,5 or 30 days were also observed. The increase in GDNF expression remained high in the dorsal root up to 30 days, but expression decreased significantly in the ventral root at 30 days after denervation. BDNF expression in the dorsal root increased steadily and remained higher than in the ventral root over time. A persistent reduction in CNTF expression in the ventral and dorsal root at all time points was also observed [95,96].

During PNS injury, regeneration-associated genes are activated, boosting the inherent neuronal growth capacity [97]. This leads to the degradation of the distal part of the axon and its associated myelin sheath by infiltrating macrophages and Schwann cells [98]. Macrophages act as phagocytes, removing myelin debris and creating a supportive regeneration microenvironment. Meanwhile, the Schwann cell provides guidance cues and secretes neurotrophic factors essential for axonal regeneration [99]. The Schwann cells dedifferentiate and proliferate to form regeneration tracks, known as bands of Büngner, which serve as physical scaffolds that guide regenerating axons across the injury site [100]. In addition to that, they secrete growth factors and extracellular matrix molecules that promote axonal growth and guide axonal navigation [101]. These cells then remyelinate the regenerating axons, restoring proper conduction of nerve impulses [102]. Additionally, intracellular signalling pathways are involved during axonal regeneration, including the cyclic AMP (cAMP) and Rho GTPase pathways, which are essential in regulating cytoskeletal dynamics and growth cones [103,104]. Moreover, the MAPK/ERK pathway is also activated concurrently with the PI3K/Akt/mTOR pathway, promoting cell survival, axonal growth, and axonal elongation [105].

Once the axon has reached the destination, the synapse will reconnect and form a new neural circuit formation. This process is mediated by a variety of molecular cues secreted by surrounding cells, which serve as chemoattractants or chemorepellents, guiding axonal growth towards or away from specific target regions and facilitating the formation of functional synapses [104,106]. For example, NCAMs and synaptic cell adhesion molecules (SynCAMs) participate in homophilic or heterophilic interactions between pre- and postsynaptic membranes, promoting synapse formation through cell adhesion [104,107]. Following target recognition, regenerating axons undergo synaptic assembly, wherein presynaptic terminals form specialised structures capable of neurotransmitter release, and postsynaptic compartments develop receptors and signalling molecules necessary for neurotransmitter reception [108]. This process involves the recruitment and clustering of synaptic proteins, including neurotransmitter vesicle proteins, synaptic scaffolding proteins, and neurotransmitter receptors at the nascent synaptic site [109]. Intracellular signalling pathways, such as those mediated by calcium/calmodulin-dependent protein kinases and Rho GTPases, regulate the dynamic organisation of synaptic components and the formation of functional synapses.

### 5.2. Central Nerve Regeneration

Regenerative capacity in the CNS is significantly diminished in comparison to that in the PNS. A crucial point to note is that oligodendrocytes, instead of Schwann cells, as in the PNS, are responsible for myelinating neuronal axons in the CNS. Some distinct characteristics between these two types of cells could explain why they have different impacts on the regeneration of the PNS and CNS [110,111]. Oligodendrocytes, which provide support and insulation to axons in the CNS, face several intrinsic and extrinsic factors that limit their proliferation and regenerative capacity following injury. Unlike Schwann cells in the PNS, oligodendrocytes exhibit a more quiescent state after maturation, and their proliferation is inhibited by several key environmental signals. The presence of inhibitory molecules in the CNS environment, such as Nogo-A, MAG, and OMgp, all contribute to a hostile milieu for axonal regeneration and oligodendrocyte function [112]. Additionally, reactive astrocytes at injury sites can produce factors like TGF-β that further inhibit oligodendrocyte proliferation and promote scar formation, which impedes neuronal regrowth [113].

Therapeutically, strategies aimed at promoting oligodendrocyte proliferation or modifying their behaviour are being explored. For instance, manipulating signalling pathways involving growth factors, such as insulin-like growth factor (IGF-1) or neurotrophins, could enhance oligodendrocyte survival and function [114]. Gene therapy approaches that inhibit inhibitory factors (e.g., using monoclonal antibodies against Nogo-A) or promote a pro-regenerative state in oligodendrocytes could also be viable strategies [115]. Furthermore, small molecules that modulate the inflammatory response or target signalling pathways related to oligodendrocyte maturation and plasticity may enhance their regenerative potential [116]. Ultimately, a combination of these approaches, alongside strategies to reduce inhibitory scar formation and promote a supportive microenvironment, could significantly facilitate axonal regeneration and improve outcomes following CNS injuries. Various approaches to promote oligodendrocyte proliferation and enhance their regenerative capacity have been tested in preclinical studies, though they have not yet been widely translated into clinical applications.

Several mechanisms have been identified as being involved in the regeneration process in the central CNS. One is canonical Wnt signalling pathways, which regulate axonal growth and remodelling in guiding axons in the developing optic nerve, brain, and spinal cord [117]. This study found that Wnt ligands, including Wnt3a, 4, and 7b, play an essential role in promoting the branching of axons, the production of growth cones, the growth of axons, and the assembly of synapses in CNS [117]. In a mouse model study, administering intravitreal injections of Wnt3a, a substance that activates the canonical Wnt/β-catenin signalling pathway, resulted in substantial development of axons beyond the site of optic nerve crush injury [118]. Furthermore, the activation of Wnt3a resulted in enhanced survival of retinal ganglion cells (RGCs) and superior functionality compared to the control group. In a different study that used rats as a model, the activation of Wnt3a signalling resulted in the proliferation of nerve fibres, improved the functioning of neurons, and stimulated the healing process following a spinal cord injury [119]. Based on their findings, Wnt3a facilitated the transformation of existing neural stem cells into neurons and stimulated the regrowth of myelin at the injury site. This resulted in the enhanced transmission of nerve impulses and improved spinal cord functioning.

Ironically, evidence also indicates that Wnt ligands that activate non-canonical Wnt-Ryk pathways can hinder the growth of axons. For instance, during the development of cortical spinal tracts and corpus callosum, the ligands Wnt1 and 5a bind to Ryk receptors and inhibit axon growth [120]. In addition, the injection of function-blocking Ryk antibodies at the site of the dorsal spinal cord injury in mice has resulted in a significant rise in the sprouting of corticospinal tract branches around the area of injury [121]. However, inhibiting the Wnt5a-Ryk signalling pathway has led to substantial development of the corticospinal tract’s axons and improved functional recovery in a rat model of spinal cord contusion [122]. Based on these findings, inhibiting non-canonical Wnt signalling has been shown to enhance axonal development and facilitate functional recovery. The Wnt5a-Ryk signalling pathway generally functions as an inhibitory mechanism in neural development and regeneration, with its suppression facilitating enhanced axonal regrowth following injury [123].

## 6. Other Nerve Regeneration Mechanisms

### 6.1. FGF Signalling Pathway

Several studies have demonstrated that FGF signalling plays a vital role during tissue repair and regeneration. The absence of Fgf2 or the simultaneous knockout of Fgfr1 and Fgfr2 in keratinocytes leads to a considerable delay in wound healing in mice [124]. Studies have shown that recombinant FGFs have the ability to enhance tissue regeneration in animal models, particularly in mice and rats [125]. The primary function of FGF during tissue repair and regeneration is to improve the wound healing process. bFGF is among the proteins that play a role in the upregulation of hepatocyte growth factor in mesenchymal cells, contributing to tissue repair [126]. These findings are supported by another study in which the ulcers treated with bFGF alone had the most effective healing, as evidenced by wound closure [127].

### 6.2. BMP Signalling Pathway

BMPs are crucial in signalling injury and coordinating glial responses and neural progenitor cell differentiation at the injury site. After CNS injury, BMPs significantly impact both neurons and glia, exerting a broad range of influences. A pro-regenerative transcription programme in neurons can be activated through BMPs, either by utilising the Smad-mediated canonical pathway or by directly influencing cytoskeletal dynamics at the growth cone through non-canonical pathways. The potential connection between the responses of the two subcellular compartments to BMPs is suggested to involve the trafficking of intra-axonal BMP signalosomes.

Primary neuron cultures demonstrate the role of BMPs as growth factors, promoting axon growth in various types of neurons, including developing striatal GABAergic neurons, raphe serotonergic neurons, cerebellar granule neurons, retinal ganglion cells (RGCs), and spinal motor neurons (SMNs) [128,129,130,131,132]. Additionally, BMP plays a crucial role in the intricate signalling network that governs axon development, interacting in association with neurotrophin signalling to ensure proper growth. For example, in dorsal root ganglia (DRG) sensory neurons, studies have found that activation of the BMP pathway promotes neurotrophins through the two effectors of RAF-MEK kinase signalling, Erk1 and Erk2 [133], that play a significant role in promoting axonal outgrowth. In addition to that, BMP-Smad1 is also involved in developmentally regulated processes and governs axonal growth in DRG neurons. Sensory axon regeneration in a mouse model of spinal cord injury (SCI) is observed when Smad1 is reactivated specifically in adult DRG neurons [134].

BMP signalling can function locally to facilitate the formation of cytoskeletal structures in distant parts of axons or dendrites, independent of Smad or non-canonical pathways. For example, the activation of LIMK occurs through its binding to type II BMPR, and it controls the process of dendritogenesis, which is dependent on BMP signalling [135]. However, in cerebellar granule neurons, BMP2 hinders the development of neurites by a process that depends on LIMK [136]. Previous studies have demonstrated that BMP7 can stimulate growth cone turning by influencing the local dynamics of actin through the activity of ADF/cofilin, which is regulated by LIMK and slingshot phosphatase [137].

### 6.3. Shh Signalling Pathway

The SHH pathway has been shown to promote the survival of axons after peripheral nerve injury [138,139,140]. Shh expression was detected in Schwann cells near the site of injury in a rat model of sciatic nerve crush, specifically on day 1 after the injury, followed by regrowing the axon within the crush zone after 7 days [140]. In addition, overall neurite outgrowth and branch formation reduction were observed when SHH knockdown was performed using siRNA [138]. A study using rats with diabetic neuropathy found that administering SHH through injections resulted in the development of new blood vessels in the sciatic nerves, along with the restoration of nerve function. These findings suggest that SHH plays a crucial role in promoting the growth of new blood vessels, which is essential for forming pathways for nerve regeneration [141].

BDNF plays a crucial role in the survival, differentiation, and protection of neural cells. It has been found to have neurodegenerative effects on sciatic and facial nerves [142,143]. In a study involving cavernous nerve crush, the levels of BDNF protein showed an initial increase of 38% on the first day, followed by a subsequent decrease of 62% below basal levels on day 4. However, by day 7 post injury, the levels returned to normal. The administration of SHH treatment resulted in a significant 36% increase in BDNF levels in normal cavernous nerves. Conversely, the inhibition of BDNF led to a notable 34% decrease in BDNF levels in normal cavernous nerves. BDNF levels showed a 44% increase in crushed cavernous nerves treated with SHH. Conversely, inhibiting BDNF in SHH-treated crushed nerves resulted in a decrease in SHH-induced nerve regeneration. These findings indicate that SHH promotes cavernous nerve regeneration by increasing the expression of BDNF [144].

## 7. Future Perspectives

The field of human neuro-regeneration, especially in relation to the CNS, is a very complex area of study that is distinguished by a few inherent limitations. Although some lower organisms are able to perform substantial neural regeneration, the human CNS shows poor ability to regenerate after an injury. This limitation comes from an internal lack of regenerative cells and the existence of inhibitory molecules that prevent nerve regeneration. One of the most notable obstacles to CNS repair is creating a glial scar triggered by astrocytes. The human body faces a multi-faceted response at the cellular level when it experiences damage to the nerve, which concerns the stimulation of the immune system to eliminate debris and damaged cells [145]. At the same time, astrocytes in the CNS become responsive and multiply, often culminating in the previously mentioned glial scar formation [30]. Damaged neurons may experience apoptosis, and while peripheral nerves try to regenerate through fibre growth, this process is usually evident in the chronic stage, especially in the CNS [146]. The inflammatory response after injury makes this landscape more difficult, dispensing cytokines and other mediators that can assist and hinder regeneration [147].

The glial scar plays a dual role in neuro-regeneration, serving both protective and inhibitory functions for neuronal regeneration [31]. The glial scar acts as a protective barrier, preventing the spread of inflammation and isolating the injury site. On the other hand, it inhibits axon regrowth by forming a dense physical and biochemical barrier composed of reactive astrocytes, microglia, and extracellular matrix molecules like chondroitin sulfate proteoglycans (CSPGs) [148]. These molecules create a hostile environment for regeneration by preventing the reformation of synaptic connections and axonal growth. Current studies are exploring several strategies to modify or manipulate glial scars to enhance their beneficial effects while limiting their inhibitory role. One promising approach involves using enzymes like chondroitinase ABC to degrade CSPGs, thereby promoting axon regrowth and neural repair [149]. Studies have shown that the enzymatic digestion of CSPGs can reduce scar-induced inhibition and restore functional connectivity in the injured spinal cord.

Another approach is targeting signalling pathways that regulate the formation of glial scars. For example, modulating the STAT3 signalling pathway in astrocytes has been found to encourage a more permissive environment for neural regeneration while maintaining the protective aspects of the scar [150]. Additionally, some researchers are exploring the use of stem cell therapy to bridge the gap created by the glial scar, providing new cells that can support regeneration and functional recovery [151]. Furthermore, researchers are developing biomaterial scaffolds and drug delivery systems for implantation at injury sites to modulate the microenvironment of scars [152]. These materials can gradually release growth factors or anti-inflammatory molecules to promote regeneration while mitigating an excessive inflammatory response. All these approaches seek to optimise the dual function of the glial scar, reducing its inhibitory impact on neural repair while preserving its essential role in curbing inflammation dissemination. These strategies aim to harness the beneficial properties of the glial scar while minimising its negative effects on regeneration, opening new avenues for treating injuries in the central nervous system.

Regeneration capacity in certain animals can effectively restore tissues and structures that were previously lost. The axolotl, polychaete, and planarian are highly regenerative animals with numerous experimental advantages due to their remarkable characteristics. These organisms are widely recognised for their remarkable ability to regenerate various body parts, such as internal organs, the CNS, and appendages, without leaving any scars, even though certain pathways, such as Wnt/β-catenin, BMP, Shh, and FGF signalling, seem to be conserved among the animal kingdoms. The neuronal diversity, complex tissue structure, and axonal connections between these organisms are still uncertain. The mammalian nervous system, especially the CNS, is known for its intricate complexity. Its capacity to perform advanced functions, such as those carried out by the cerebral cortex, depends on the existence of a wide range of specialised types of neurons. These neurons are interconnected through both long-distance and local circuits, all functioning within a well-defined tissue structure. The restoration of higher-order CNS structures will probably involve the regeneration of various neuronal subtypes, the reconstruction of original connections, and the integration of newborn neurons into the existing tissue. The extent to which regenerative species can accomplish complex tasks is still unknown, apart from their general ability to generate new neurons and rebuild the overall structure of the brain.

An expanding body of research encompassing preclinical trials on tissue-engineered nerve grafts derived from stem cells sheds light on the promising prospects for using stem cells in neuro-regeneration (Table 1). Stem cell therapies have become a potential therapeutic tool for regenerating nerve cells [153]. The progress of treatments influencing neurotrophic aspects, which support nerve growth and survival, illustrates another critical advancement. Significant advancements have been made in the interaction between stem cells and nanostructures or nanomaterials, especially in scaffolds that assist nerve growth by imitating the extracellular matrix [154]. Furthermore, gene therapy that manipulates or regulates crucial nerve regeneration genes has exhibited significant neuro-regeneration potential by integrating gene and cell therapy to create an ideal milieu for the regeneration of axons and the restoration of functional circuits [155]. Lastly, implementing immunomodulation strategies aimed at adapting the immune system’s responses to injury and synchronising the local inflammatory response have considerable importance in restoring peripheral nerve injury (PNI) [156].

Future study on neuro-regeneration should prioritise both the morphological restoration of CNS structures and the long-term functional recovery of intricate CNS processes, including cognition, motor coordination, and sensory perception. To ensure this, experimental models must incorporate functional evaluations in conjunction with structural analysis. Animal models, particularly rodents such as mice and rats, are extensively employed to assess cognitive and motor recovery via behavioural tests, including the Morris water maze for spatial memory, the rotarod test for motor coordination, and somatosensory testing for sensory perception. To enhance translatability to humans, more sophisticated models such as non-human primates or organoids can yield significant insights into higher-order cognitive functions and human-specific brain responses. Cerebral organoids can replicate human brain development and pathology, providing a distinctive platform to investigate the interaction between neural regeneration and functional recovery in a regulated setting.

It is crucial to construct rigorous, long-term clinical trials that assess not just morphological results, such as axonal regeneration by imaging techniques (e.g., MRI, DTI), but also neurophysiological and behavioural outcomes. Clinical trials ought to include modalities such as electroencephalography (EEG) or functional magnetic resonance imaging (fMRI) to evaluate cerebral activity and track the recuperation of cognitive functions longitudinally. Additionally, examinations such as the Mini-Mental State Examination (MMSE) or motor function evaluations (e.g., the 9-Hole Peg Test for coordination) might yield significant information regarding patients’ functional recovery. Longitudinal studies are essential to track enduring changes, confirming that early anatomical success results in persistent functional benefits, hence facilitating the advancement of treatment techniques for CNS regeneration.

Nevertheless, the effectiveness of neuro-regeneration in humans is very much connected to elements such as age and current health conditions. The ability to regenerate usually diminishes with age, caused by decreased cell proliferation and an augmented inflammatory response. Chronic diseases like diabetes or vascular disorders can disrupt blood flow and nutrient supply, which dangerously interferes with regenerative processes. Genetic inclinations also affect specific disorders, influencing the body’s regenerative abilities. Lifestyle factors such as diet, exercise, and hormonal changes, especially those related to ageing, heavily impact the body’s ability to regenerate.

## 8. Conclusions

Understanding neuro-regeneration mechanisms from these organisms (axolotls, polychaetes, and planarians) holds immense potential, particularly in neuro-regeneration. These model organisms offer unique advantages for researchers, allowing them to explore the intricacies of nerve cell regeneration, neural circuit reconstruction, and the role of stem cells in the process. As scientists unravel the mysteries of neuro-regeneration in these organisms, the hope is that this knowledge will pave the way for developing therapeutic strategies to enhance the limited regenerative capacity of the human nervous system. The journey to unlocking the full potential of neuro-regeneration is ongoing, and continued research in this field holds promise for ground-breaking advancements in neurological medicine.

## Figures and Tables

**Table 1:** List of peripheral nerve regeneration treatments.

Type	Mechanism of Action	References
Nerve Graft		
Nerve Autografts	Nerve autografts are typically obtained from autologous tissues, such as nerves, arteries, and muscles. The current preferred method for repairing peripheral nerve injuries is to replace the damaged nerves with nerve autografts. This approach is regarded as the gold standard since it reduces immune reactions and creates an optimal environment for nerve regeneration, leading to a therapeutic outcome.	[157]
Nerve Allografts	Nerve allograft transplantation is indicated for the treatment of severe peripheral nerve injuries that are not amenable to conventional methods such as autologous nerve grafting or nerve transfer procedures.	[158]
Pharmacotherapy		
Erythropoietin (EPO)	EPO is speculated to be synthesised by perineuronal Schwann cells in the peripheral nervous system. Its production is triggered by the upregulation of calcitonin gene-related peptide (CGRP), as well as the activation of signalling pathways such as PI3K/Akt, JAK/STAT-3, and NF-kB. These pathways play a role in promoting the survival of neurons.	[159]
Tacrolimus (FK506)	Tacrolimus is an immunosuppressant which acts by blocking the activity of calcineurin. The binding of FK506 to heat shock protein 90 (Hsp90) and protein 52 (FKBP-52) and the activation of the ERK pathway have effects on nerve regeneration.	[160]
N-acetylcysteine (NAC)	NAC functions involve providing cysteine for the generation of glutathione, and it has the ability to inhibit apoptotic signalling in neuronal cells through the RAS-ERK pathway. NAC induces an increase in the expression of Bcl-2 while simultaneously causing a decrease in the mRNA levels of pro-apoptotic proteins, including caspase-3 and Bax.	[161]
Geldanamycin	Geldanamycin exhibits high affinity for Hsp90 and has been demonstrated to enhance the speed of axon regeneration and facilitate early restoration of motor neuron function following tibial nerve crush injury.	[162]
Stem Cell Therapy		
Embryonic Stem Cell	Embryonic stem cells that undergo differentiation into Schwann cells exhibit the presence of Schwann cell markers (S100 and p75). Additionally, they stimulate the process of myelination in neurons located in the dorsal root ganglia. Injecting neural progenitor cells generated from murine embryonic stem cells into the epineurium of transected sciatic nerves in rats resulted in significant improvement in both function and structure.	[163]
Bone Marrow Mesenchymal Stem Cell (BMMSC)	BMMSCs have the ability to differentiate into Schwann-like cells, which in turn promotes the growth of neurites. The introduction of BMMSCs into silk fibroin-based nerve conduits resulted in an increase in the levels of Schwann cell markers (S100). This increase subsequently led to the elevation of various growth factors, ultimately expediting the recovery process in murine models with sciatic nerve injuries.	[164]
Adipose Stem Cell (ASC)	ASCs have the ability to differentiate into spindle cells that can exhibit markers of Schwann cells and have the capacity to promote the development of myelin sheaths.	[165]
Nerve Conduits		
Polyglycolic acid (PGA)	PGA facilitated the utilisation of materials that were more flexible and porous, resulting in an improved process of nerve regeneration. PGA nerve conduits proved to be more effective in reconstructing long nerve gaps when compared to alternative synthetic polymer materials. The efficacy of PLA nerve conduits promotes neuron proliferation and enhances axon maturation.	[166]
Poly(L-lactide-co-ε-caprolactone) (PLCL)	PLCL conduits have the potential to generate lower levels of lactic acid as they degrade, making them a more practical choice due to their decreased toxicity. The effectiveness of this conduit has been evaluated with varying results. Studies indicate that PLCL does not demonstrate significant efficacy in promoting nerve repair in digital nerves. However, it has shown comparable outcomes in end-to-end repair.	[167]
Collagen	Collagen has been widely employed in the fabrication of nerve conduits due to its low biodegradability and immunogenicity. The material most commonly utilised is Type I collagen, which forms fibrils of the endoneurium in the basal lamina.	[168]
